# The elusive biomarker

**DOI:** 10.1038/s41390-022-02247-w

**Published:** 2022-08-18

**Authors:** Simone Huntingford, Rod W. Hunt

**Affiliations:** 1 Monash Newborn, Monash Health, Clayton, VIC Australia; 2 Department of Paediatrics, Monash University, Clayton, VIC Australia

For many years now, Neonatologists and researchers have interrogated the validity of various potential biomarkers to predict neurodevelopmental outcomes in neonates born prematurely. Clinically, a robust biomarker may help to identify at-risk infants and drive referral to early intervention services. At the bedside, the information might be used to prepare families for their child’s prognosis. In the research setting, a reliable biomarker may serve as a useful short-term indicator of an intervention’s impact. The investigation of candidate biomarkers has spanned across many different aspects of perinatal medicine. Perinatal risk factors, early developmental assessments, neuroimaging, and social and environmental factors have all been explored.^1,2^ Attempts have been made to identify single parameter predictors, such as Apgar scores,^3,4^ while other approaches have combined makers to create robust predictors of early risk, such as the Score for Neonatal Acute Physiology (SNAP) and the Clinical Risk Index for Babies with their respective variations.^5^ Despite years of research, the quest to better prognosticate neurodevelopmental outcomes for many preterm neonates remains challenging.

In their recent study, Gervais et al. used a retrospective cohort design to investigate the association between sodium fluctuations in very preterm neonates and the composite outcome of death or neurodevelopmental impairment at 18 months corrected age.^6^ The study followed 147 consecutive neonates born <29 weeks’ gestation recruited from the Newborn Intensive Care Unit (NICU). Sodium values were obtained for the first 30 days of life, which revealed that the sodium fluctuations were greatest between day 1 and day 10; the latter interval forming the focus of the analysis. The study team was able to capture neurodevelopmental data at 18 months corrected age for 85% of the cohort, a highly commendable effort in the midst of a global pandemic. The major finding was that higher fluctuations in glucose-corrected plasma sodium were associated with death or neurodevelopmental impairment at 18 months corrected age. Higher fluctuations in plasma sodium were noted in infants treated with non-steroidal anti-inflammatory drugs (NSAIDs).

Do sodium fluctuations simply reflect the degree of underlying illness? Unwell neonates have numerous risk factors for fluctuations in fluid status and sodium levels. Neonates, particularly those born preterm, have physiologically lower glomerular filtration rates, rendering them susceptible to stressors, such as sepsis, hypoxia and hypotension.^7^ Poor urinary concentrating ability is combined with high insensible losses.^7^ Medication delivery may require the coadministration of fluid, sodium or glucose, and nephrotoxic agents (such as aminoglycosides and NSAIDS) are commonly used in the NICU. Gervais et al. collected data on variables of interest, including gestational age, presence and severity of bronchopulmonary dysplasia (BPD), SNAP score, NSAID use, and acute kidney injury (AKI). More severe sodium fluctuations were associated with SNAP score and BPD; however, these associations did not persist when corrected for gestational age. The association between higher sodium fluctuations and death or neurodevelopmental impairment remained significant when corrected for either gestational age or SNAP score.

An association between sodium fluctuations and neurodevelopmental outcome had already been demonstrated by Baraton et al.^8^ Their study included very preterm infants born <33 weeks’ gestation and demonstrated that greater changes in sodium levels were significantly associated with the risk of impaired functional outcomes at 2 years corrected age. Differentiating from this study design, Gervais et al. incorporated the Katz formula^9^ to account for plasma glucose levels. This was a pertinent element of the study design given the effects of glucose levels on both plasma osmolality and on preterm neurodevelopmental outcomes.^10^

Gervais et al. also importantly reported a significant association between sodium fluctuations and severe intraventricular haemorrhage (IVH), independent of gestational age. Sodium fluctuation is a targeted parameter nested within the context of neonatal renal function. Other groups have sought to establish associations between renal dysfunction and neurological injury. A related association between AKI and IVH was demonstrated using data from The Assessment of Worldwide Acute Kidney Epidemiology in Neonates cohort. The cohort comprised 825 eligible infants <33 weeks’ gestation. After controlling for parameters of clinical instability, infants with AKI had a 1.6 times higher adjusted odds ratio to develop any grade IVH that was statistically significant.^11^ Mountasser et al. demonstrated an association between acute kidney injury and radiological brain injury on term-equivalent brain magnetic resonance imaging in very preterm infants.^12^ Comparing the groups with and without AKI, the study found no significant difference in demographics and a range of clinical characteristics. Gervais et al. along with these studies, add to the evolving picture that neurodevelopmental outcome depends on an interplay of factors from multiple systems. It seems that the neonatal kidney is not simply a bystander in the neurodevelopmental landscape.

The authors have summarised some of the limitations of their study, such as a proportion of infants with absent data for neurodevelopmental outcome (15%) and NSAID use (15%) and the inability to accurately quantify electrolyte administration from the retrospective data set. The latter is particularly of interest. Due to their low body mass, neonates may receive relatively substantial quantities of fluid and electrolytes via catheter flushes, line patency infusions, medication infusions and medication carrier mediums. However, previous studies have reported that preterm neonates also commonly receive fluid boluses.^13^ Of evolving concern is the link between anti-hypertensive therapy (including fluid boluses) and death and adverse neurodevelopmental outcome in preterm neonates.^13^ In the NICU, clinicians frequently balance the perceived benefits of fluid bolus therapy on systemic and cerebral perfusion, with the risk of rapid fluid shifts endangering the delicate vasculature of the preterm brain. It would be interesting to understand more about the impacts of fluid bolus therapy on sodium fluctuations and tease out the relative contributions of each parameter on neurodevelopmental outcome. Gervais et al. have certainly provided the groundwork for a number of future research directions.

What can we translate from this new study? Sodium dyshomeostasis is a common phenomenon in the NICU despite best efforts to achieve balance. Given that the evolving evidence points to much broader consequences than commonly appreciated, it may be pertinent to turn our attention to the neonatal kidney. Renoprotective strategies could be implemented, including more judicious use of medications (such as NSAIDS and aminoglycosides) that risk kidney injury and altered sodium handling. Careful management of fluid, sodium and glucose delivery may avoid some of the fluctuations described in Gervais’ cohort. The effects of better defined strategies on plasma sodium and its associated neurodevelopmental outcomes remain to be seen.
